# Early Recurrence of Cardiac Myxomas in an Adolescent With Carney Complex: Clinical Clues Preceding Imaging Findings—A Case Report

**DOI:** 10.1155/cric/7261880

**Published:** 2026-05-25

**Authors:** Bastian Abarca-Rozas, Francisco Saavedra, Juan Pablo Retamal, Francisco Vergara, Javier Revello, Pamela Zelada, Demian Andrés Fullerton

**Affiliations:** ^1^ Internal Medicine Unit, Department of Internal Medicine, Sótero del Río Hospital, Santiago, Chile; ^2^ Coronary Care Unit and Cardiovascular Intensive Care Unit, Department of Cardiology, Sótero del Río Hospital, Santiago, Chile; ^3^ Cardiovascular Surgery Unit, Department of Cardiology, Sótero del Río Hospital, Santiago, Chile; ^4^ Department of Pediatric Cardiology, Sótero del Río Hospital, Santiago, Chile

**Keywords:** cardiac myxoma, cardiology, Carney complex, case report, tumor recurrence

## Abstract

**Background:**

Carney complex is a rare autosomal dominant syndrome characterized by multiple cardiac myxomas with early onset and high recurrence rates, associated with a significant risk of intracardiac obstruction and embolic events.

**Case Presentation:**

A female patient was diagnosed at age 14 with multiple intracardiac masses involving both atria and the right ventricle, which were confirmed as myxomas and surgically resected. She also presented with mucocutaneous lentiginosis and a pituitary microadenoma, establishing a clinical diagnosis of Carney complex despite negative genetic testing. During follow‐up, new masses were initially interpreted as intracardiac thrombi and treated with anticoagulation but were subsequently reclassified as tumor recurrence based on multimodal imaging. Progressive tumor burden led to a second surgical intervention with complete resection of multicameral lesions, with favorable clinical evolution.

**Conclusion:**

This case illustrates the systemic and recurrent nature of Carney complex and highlights the critical role of cardiac magnetic resonance imaging in differentiating thrombus from tumor, optimizing therapeutic decision‐making, and avoiding unnecessary interventions.

## 1. Introduction

Carney complex (CNC) is a rare hereditary syndrome with autosomal dominant inheritance and variable penetrance, characterized by the association of cardiac, endocrine, and cutaneous tumors, along with typical pigmentary manifestations such as mucocutaneous lentiginosis [1, 2]. Among its clinical features, cardiac myxomas represent the main determinant of morbidity and mortality due to their obstructive and embolic potential, as well as their distinctive biological behavior, characterized by early onset, multicameral involvement, and a high rate of recurrence following surgical resection [3–5].

In contrast to sporadic myxomas—typically solitary, located in the left atrium, and associated with a low recurrence rate—CNC‐associated myxomas reflect a systemic tumor predisposition, with a tendency toward multiple lesions, true recurrences, and de novo tumor development in different cardiac chambers [4, 5]. This pattern poses significant clinical challenges, including the need for long‐term surveillance, decision‐making regarding the optimal timing of reintervention, and, particularly, the differentiation between tumor recurrence and intracardiac thrombus in previously operated patients. In this context, tissue characterization using multimodal imaging, especially cardiac magnetic resonance, plays a central role in the evaluation of intracardiac masses, guiding diagnosis and preventing potentially unnecessary therapeutic interventions [4, 5].

We present the case of a young patient with a clinical diagnosis of CNC based on phenotypic criteria despite the absence of genetic confirmation, whose course was characterized by multicameral cardiac involvement and multiple recurrences after surgical resection. This case illustrates the dynamic and systemic nature of the disease and provides clinically relevant evidence regarding the complexity of the differential diagnosis of intracardiac masses in this setting, as well as the challenges in therapeutic decision‐making and long‐term follow‐up.

## 2. Case Presentation

**Figure 1:**
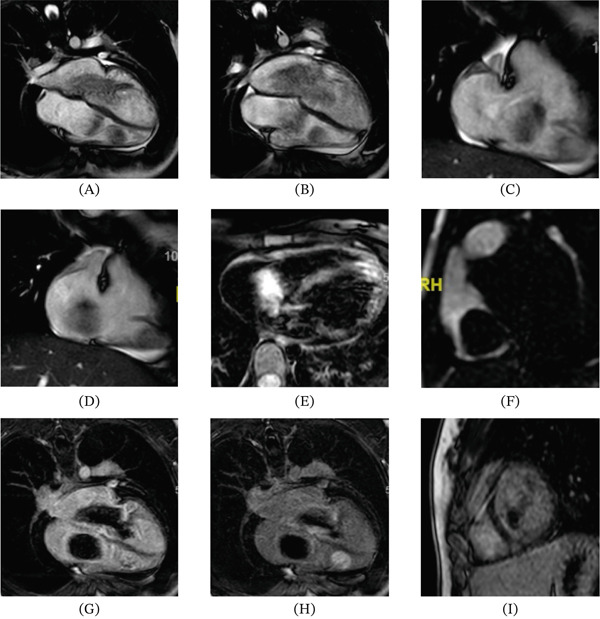
Cardiac magnetic resonance imaging demonstrating multiple intracardiac masses: (A, B) four‐chamber long‐axis cine images showing mobile right and left atrial masses with protrusion through the atrioventricular valves. (C, D) Right ventricular long‐axis cine images demonstrating a third mass attached to the mid–apical free wall of the right ventricle. (E) Fat‐suppressed T2‐weighted sequence showing hyperintensity of the masses. (F) First‐pass perfusion image showing no contrast uptake by the masses. (G) Early gadolinium enhancement image demonstrating peripheral enhancement of the left atrial tumor. (H) Late gadolinium enhancement atrial‐level image showing enhancement of the left atrial tumor, with no enhancement of the right atrial tumor. (I) Late gadolinium enhancement image demonstrating enhancement of the right ventricular tumor.

A female patient with a history of mild asthma and previously good general health presented at age 14 with constitutional symptoms, mild exertional dyspnea, and the recent detection of a cardiac murmur. Initial transthoracic echocardiography revealed multiple intracardiac masses located in the right atrium, left atrium, and right ventricle. Cardiac magnetic resonance imaging confirmed lesions consistent with myxomas, demonstrating multicameral involvement and tissue characteristics compatible with CNC (Figure 1).

**Figure 2:**
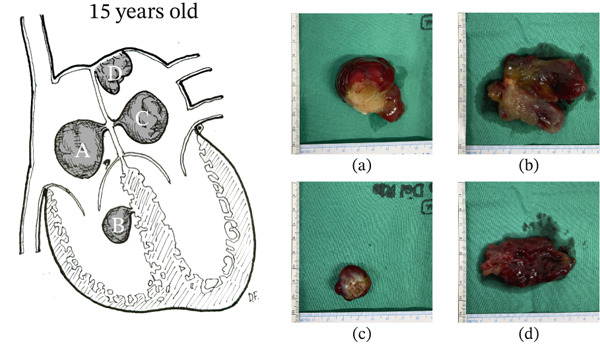
Tumors at 15 years of age. Schematic representation of the location of myxomas resected at age 15, alongside photographs of the excised specimens. Illustration by Demian Fullerton.

Given the potential risk of valvular obstruction and embolic events associated with the number and location of the lesions, cardiac surgery was performed at age 15, with resection of four intracardiac masses. The anatomical distribution of the lesions and the resected specimens are detailed in Figure 2. The patient had a favorable immediate postoperative course, with restoration of ventricular function and resolution of symptoms. Histopathological examination demonstrated proliferation of stellate and polygonal cells arranged in cords and perivascular structures, embedded in abundant myxoid stroma with hemorrhagic foci, consistent with cardiac myxoma. The lesions involved the right atrium, left atrium—including the interatrial septum and superior wall—and the right ventricle, confirming a multifocal pattern. The pathology report did not specify surgical margin status. In the absence of evidence of intracardiac thrombosis, anticoagulation was not initiated postoperatively.

During this period, systemic evaluation revealed mucocutaneous lentiginosis, a small pituitary microadenoma, and a benign thyroid nodule. Endocrine evaluation documented mild hyperprolactinemia attributed to the microadenoma, without clinically relevant thyroid or adrenal dysfunction. Genetic testing was negative for mutations associated with CNC, including AIP, CDKN1B, GNAS, MEN1, and PRKAR1A. In subsequent years, the patient remained clinically stable, with no evidence of residual masses on serial echocardiography and preserved ventricular function.

Later, during evaluation for abnormal uterine bleeding, computed tomography identified a new intracardiac mass. Given the prior surgical history, the finding was initially interpreted as intracardiac thrombus, and treatment with aspirin and acenocoumarol was initiated. However, reevaluation with echocardiography and cardiac magnetic resonance revealed tissue characteristics inconsistent with thrombus and suggestive of myxomatous tissue. In this context, the diagnosis of thrombus was excluded, and anticoagulation was discontinued, with no subsequent embolic events.

Subsequent echocardiographic follow‐up identified new intracardiac lesions, including masses in the left atrium and right ventricle. The right ventricular lesion was initially managed conservatively due to stability in size, absence of hemodynamic compromise, and lack of attributable symptoms. Similarly, the left atrial lesion was observed in the absence of significant valvular obstruction or embolic events. However, serial follow‐up demonstrated progressive increase in tumor burden, associated with persistent fatigability and episodes of precordial pain. Cardiac computed tomography angiography confirmed three intracardiac masses: two in the left atrium and one in the right ventricle, including involvement of the atrial septum.

**Figure 3:**
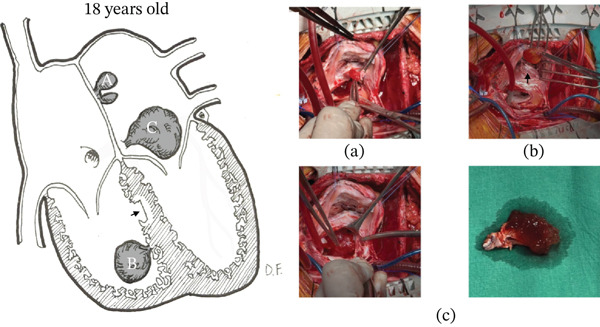
Second resection with coronary sinus unroofing. Schematic representation of the location of myxomas resected at age 18. (a) recurrence at the interatrial septal scar; (b) de novo tumor in the left atrium along the course of the coronary sinus; (c) de novo tumor arising from the interventricular septum, distant from the previous scar. Illustration by Demian Fullerton.

**Figure 4:**
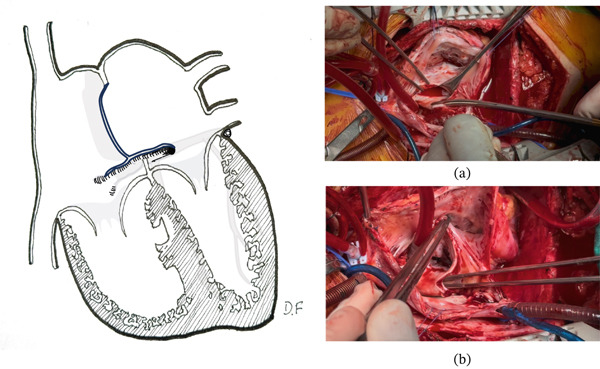
Second resection with coronary sinus unroofing. The reoperation included resection of the interatrial septum, unroofing of the coronary sinus, and partial resection of the interventricular septum. (a) Intraoperative photograph showing resection of the tumor by cutting the coronary sinus and the overlying left atrial wall using a transseptal approach to the left atrium. (b) The coronary sinus after unroofing, with a forceps inserted into its lumen and the mitral valve visualized in the open position. Illustration by Demian Fullerton.

**Figure 5:**
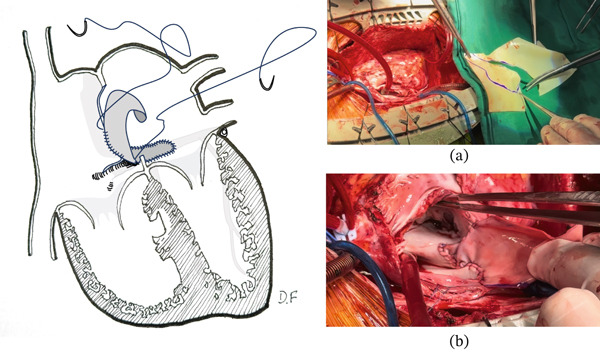
Repair of the coronary sinus and atrial septal defect. The coronary sinus was repaired using a bovine pericardial patch shaped like a paddle. The narrower section was used to reconstruct the roof of the coronary sinus, while the wider section was used to repair the interatrial septum. Illustration by Demian Fullerton.

Given the potential obstructive and embolic risk—particularly due to left atrial location in relation to mitral inflow—a second surgical intervention was performed in early adulthood. Intraoperative findings revealed three lesions (Figure 3): a recurrence associated with the atrial septal scar, a de novo lesion on the floor of the left atrium along the course of the coronary sinus causing partial obstruction of mitral flow, and a de novo lesion in the right ventricle arising from the interventricular septum. Resection included wide excision of the interatrial septum and unroofing of the coronary sinus to allow adequate tumor exposure (Figure 4). Reconstruction was performed using a bovine pericardial patch, restoring continuity of the septum and coronary sinus (Figure 5). Right ventricular resection included the tumor pedicle with a margin of surrounding myocardium, achieving macroscopically complete excision. The postoperative course was favorable, with no major complications, except for subclinical first‐degree atrioventricular block. Histopathology confirmed cardiac myxomas without evidence of malignant transformation, and surgical margins were free of disease. In the absence of intracardiac thrombosis, anticoagulation was not initiated. Early follow‐up showed clinical stability without evidence of early recurrence.

## 3. Discussion

This case paradigmatically illustrates the clinical phenotype of CNC‐associated cardiac myxomas, characterized by early onset, multicameral involvement, and high recurrence rates, in contrast to sporadic myxomas, which are typically solitary, left atrial, and associated with low recurrence risk [1, 3, 4].

Adolescent onset with simultaneous involvement of both atria and the right ventricle represents a pattern highly suggestive of CNC and is associated with increased tumor burden and recurrence risk. According to Stratakis diagnostic criteria, the presence of two major criteria—multiple cardiac myxomas and mucocutaneous lentiginosis—is sufficient to establish the diagnosis of CNC, even in the absence of genetic confirmation. In this case, the diagnosis was established clinically based on these criteria [1, 2]. This is particularly relevant, as a nonnegligible proportion of patients lack identifiable mutations in PRKAR1A or other associated genes, reinforcing the importance of phenotypic recognition in clinical practice.

Systemic evaluation revealed extracardiac manifestations consistent with the CNC spectrum, including mucocutaneous lentiginosis and a pituitary microadenoma with mild hyperprolactinemia. Although endocrine abnormalities were present, they were not considered sufficient to explain the patient′s fatigability, which was primarily attributed to intracardiac tumor burden. This finding suggests a multifactorial origin of symptoms and underscores the need for integrated clinical assessment in multisystem diseases.

From a pathophysiological perspective, the observed recurrence pattern—including lesions at previously operated sites and in new locations—suggests a systemic neoplastic process rather than purely local recurrence due to incomplete resection. This is consistent with CNC biology, in which tumor predisposition drives both de novo lesion development and true recurrences [3, 4]. In this context, the absence of information regarding surgical margins from the first intervention limits precise differentiation between mechanisms; however, the subsequent anatomical distribution supports a predominantly systemic component.

A critical aspect of this case was the differentiation between intracardiac thrombus and tumor recurrence, a complex diagnostic scenario in patients with prior cardiac surgery. In this case, initial computed tomography suggested thrombus, prompting empirical anticoagulation. However, no histological confirmation was obtained, and diagnostic reclassification was based on integration of multimodal imaging findings—particularly echocardiography and cardiac magnetic resonance—along with clinical evolution, demonstrating features inconsistent with thrombus and suggestive of myxomatous tissue. This approach highlights the value of noninvasive tissue characterization in the evaluation of intracardiac masses and its relevance in scenarios of diagnostic uncertainty [4, 5].

From a management perspective, this case reflects the complexity of therapeutic decision‐making in CNC patients. Initial conservative management of stable lesions without hemodynamic compromise or embolic events is consistent with an individualized strategy aimed at balancing cumulative surgical risk against the inherent risk of tumor lesions. However, a progressive increase in tumor burden and persistent symptoms justified a second intervention. This approach aligns with available evidence suggesting surgical treatment in the presence of tumor growth, valvular obstruction, embolic risk, or attributable symptoms [1, 3, 5].

Surgically, reintervention required a more aggressive approach due to anatomical complexity and lesion location. Wide resection of the interatrial septum and unroofing of the coronary sinus allowed adequate tumor exposure in a difficult‐to‐access region. It is important to note that this technique does not directly reduce recurrence but rather facilitates complete exposure of the tumor implantation base, enabling wider resection of the pedicle in anatomically complex sites. Thus, it indirectly contributes to reducing the risk of persistence or local recurrence by optimizing surgical radicality [6].

A clinically relevant observation is the retrospective identification of fatigability as a sentinel symptom preceding both surgical interventions. This finding underscores the importance of integrating nonspecific symptoms into longitudinal surveillance of CNC patients, as they may precede structural progression detectable by imaging. In this context, symptoms should not be attributed solely to mild comorbidities, particularly in patients at high risk of tumor recurrence.

Finally, the multisystem nature of CNC necessitates multidisciplinary follow‐up, integrating cardiological, endocrinological, and dermatological evaluation. Extracardiac manifestations, although clinically mild in this case, represent key elements for both diagnosis and follow‐up and may indirectly influence clinical evolution and adherence to long‐term monitoring [2, 3].

## 4. Conclusion

This case illustrates the recurrent, multicameral, and clinically unpredictable nature of cardiac myxomas in young patients with CNC, highlighting the coexistence of recurrent and de novo lesions even after apparently complete surgical resections. Differentiation between intracardiac thrombus and tumor recurrence represents a critical diagnostic challenge with direct therapeutic implications, in which multimodal imaging—particularly cardiac magnetic resonance—plays a central role in guiding clinical decision‐making and avoiding unnecessary treatments.

The observed course, with subtle symptoms preceding structural findings, reinforces the importance of systematically integrating clinical changes into longitudinal surveillance. In this context, management requires an individualized strategy based on the integration of clinical and imaging findings, with dynamic thresholds for surgical indication.

Overall, this case underscores the need for prolonged and multidisciplinary follow‐up in patients at high risk of recurrence and highlights that the absence of genetic confirmation does not exclude the diagnosis nor modify the clinical behavior of the disease. It also reinforces the value of multimodal evaluation in decision‐making and the need to maintain a high index of suspicion for new intracardiac masses in this patient population.

## Funding

No funding was received for this manuscript.

## Ethics Statement

This study was conducted in accordance with the Declaration of Helsinki and current Chilean legislation (Law No. 19,628). Ethical approval was waived by the institutional review board due to the nature of a single case report.

## Consent

Written informed consent for publication of this case report and the accompanying images was obtained from the patient′s legal guardians. All data were anonymized to protect the patient′s identity.

## Conflicts of Interest

The authors declare no conflicts of interest.

## Data Availability

The data supporting this case report are not publicly available due to patient privacy considerations but are available from the corresponding author upon reasonable request.
